# Boolean modeling and fault diagnosis in oxidative stress response

**DOI:** 10.1186/1471-2164-13-S6-S4

**Published:** 2012-10-26

**Authors:** Sriram Sridharan, Ritwik Layek, Aniruddha Datta, Jijayanagaram Venkatraj

**Affiliations:** 1 Texas A & M University, Electrical and Computer Engineering, College Station, TX, 77843-3128, USA; 2 Texas A & M University, Vet Integrative Biosciences, College Station, TX, 77843-4458, USA

## Abstract

**Background:**

Oxidative stress is a consequence of normal and abnormal cellular metabolism and is linked to the development of human diseases. The effective functioning of the pathway responding to oxidative stress protects the cellular DNA against oxidative damage; conversely the failure of the oxidative stress response mechanism can induce aberrant cellular behavior leading to diseases such as neurodegenerative disorders and cancer. Thus, understanding the normal signaling present in oxidative stress response pathways and determining possible signaling alterations leading to disease could provide us with useful pointers for therapeutic purposes. Using knowledge of oxidative stress response pathways from the literature, we developed a Boolean network model whose simulated behavior is consistent with earlier experimental observations from the literature. Concatenating the oxidative stress response pathways with the *PI*3-*Kinase-Akt *pathway, the oxidative stress is linked to the phenotype of apoptosis, once again through a Boolean network model. Furthermore, we present an approach for pinpointing possible fault locations by using temporal variations in the oxidative stress input and observing the resulting deviations in the apoptotic signature from the normally predicted pathway. Such an approach could potentially form the basis for designing more effective combination therapies against complex diseases such as cancer.

**Results:**

In this paper, we have developed a Boolean network model for the oxidative stress response. This model was developed based on pathway information from the current literature pertaining to oxidative stress. Where applicable, the behaviour predicted by the model is in agreement with experimental observations from the published literature. We have also linked the oxidative stress response to the phenomenon of apoptosis via the *PI*3*k/Akt *pathway.

**Conclusions:**

It is our hope that some of the additional predictions here, such as those pertaining to the oscillatory behaviour of certain genes in the presence of oxidative stress, will be experimentally validated in the near future. Of course, it should be pointed out that the theoretical procedure presented here for pinpointing fault locations in a biological network with feedback will need to be further simplified before it can be even considered for practical biological validation.

## Introduction

The control of gene expression in eukaryotic organisms is achieved via *multivariate *interactions between different biological molecules such as proteins and DNA [1]. Consequently, in recent years, various genetic regulatory network modeling approaches such as differential equations and their discrete-time counterparts, Bayesian networks, Boolean networks (BNs) and their probabilistic generalizations, the so-called probabilistic Boolean networks (PBNs) [2] have been proposed for capturing the holistic behavior of the relevant genes. Some of these approaches such as differential equations involve finer models and require a lot of data for inference while others such as Boolean networks yield coarse models with lower data requirements for model inference. On the other hand, historically biologists have focused on experimentally establishing marginal cause-effect relationships between different pairs of genes, which when concatenated together leads to what is known as *pathway *information. Biological pathways are used by biologists to represent complex interactions occurring at the molecular level inside living cells [3]. Pathway diagrams describe how the biological molecules interact to achieve their biological function in the presence of appropriate stimuli [4]. At a very simple level, biological pathways represent the graphical interactions between different molecules. However, as already noted, the pathways give only a marginal picture of the regulations (up-regulation or down-regulation) of the different genes/RNAs/proteins by other genes/RNAs/proteins.

The complexity of biological signaling and the prevelance of prior information in the form of pathway knowledge demand that genetic regulatory network models consistent with pathway information be developed. Motivated by this, we developed an approach to generate Boolean network models consistent with given pathway information and applied it to studying the p53-mediated DNA damage stress response [5]. In addition, we used a signaling diagram of the *MAP-Kinase *pathways to predict possible location(s) of the single signaling breakdowns, based on the cancer-causing breakdown signature [6]. Moreover, we also made *theoretical *predictions of the efficacy of different combination therapies involving six anti-cancer drugs, which we plan to validate in the near future.

In this paper, we first develop a Boolean network model consistent with oxidative stress response pathway information from the biological literature. Thereafter this model is linked with the *PI*3*k/Akt *pathway and the composite model is used to pinpoint the possible fault locations based on the observed deviations in the apoptotic signature over different time windows. The paper is organized as follows. Section contains a brief general description of Stress Response Pathways while Section presents a discussion specific to the case of oxidative stress. The Boolean network model for oxidative stress response is developed in Section. The role of mitochondria as the site in a cell where the oxidative stress is generated is discussed in Section. In Section, we develop an integrated network linking oxidative stress response to the phenomenon of apoptosis via the *PI*3*k/Akt *pathways. Section presents an approach for pinpointing fault locations in the integrated network by observing the apoptotic signature in response to certain test stress input sequences. Finally, Section contains some concluding remarks.

### Stress response pathways

**Figure 1:**
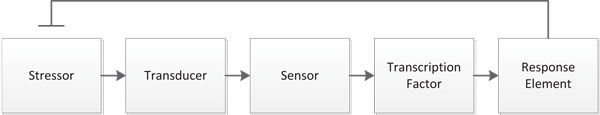
**General scheme of stress response pathways**. This figure explains the general flow of information in stress response pathways.

Adaptive stress response pathways are the first responders to chemical toxicity, radiation, and physical insults. The different stress response pathways share a very similar architecture. This architecture has three main components: a transducer, a sensor and a transcription factor (TF) [7]. The transcription factor (TF) is a DNA-binding protein that interacts with the promoter regions of its target genes via its canonical DNA-binding sites, known as 'response elements' (REs), to activate the expression of the target genes. The sensor is a protein that physically interacts with the transcription factor in the cytosol, sequestering the transcription factor from the nucleus under normal cellular conditions. In addition to its role in cytoplasmic sequestration of the TF, the sensor may direct TF degradation, providing an additional layer of regulatory control. The result of the sensor-TF complexation is to maintain inactivity of the TF under normal cellular conditions, while providing a mechanism that permits activation in response to an appropriate insult to the cell. The transducer is an enzymatic protein, such as a kinase, that conveys a biochemical change from a signaling pathway upstream of the sensor/TF complex in the event of cellular stress. The transducer may directly modify the transcription factor, providing the activating signal or modify the sensor which in turn, destabilizes the sensor/TF complex. Liberated, stabilized, and activated, the transcription factor relocates to the nucleus where it activates its target genes. Generally the sensor and TF are unique for a given stress response pathway unlike transducers which can be shared between different stress response pathways, leading to what is commonly referred to as 'crosstalk' between the pathways. A schematic diagram showing the general architecture of a stress response pathway is shown in Figure 1.

### Oxidative stress response pathways

**Figure 2:**
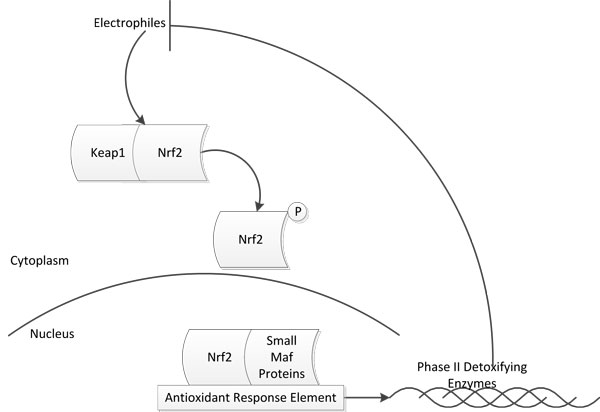
**Nrf2 Activation**. Explains how Nrf2 is activated and how it is able to neutralize the free radicals.

Oxidative stress is caused by exposure to reactive oxygen intermediaries/species (*ROS*). The stress induced on the cells by electrophiles and oxidants gives rise to a variety of chronic diseases. The outcome of interactions between the cell and oxidants is determined largely by the balance between the enzymes that activate the reactive intermediaries and the enzymes that detoxify these reactive intermediaries [8]. For example, oxidative stress contributes to aging and age-related diseases such as cancer, cardiovascular disease, chronic inflammation, and neurodegenerative disorders. The body has developed a variety of counteractive measures for combating oxidative stress. At elevated concentrations of electrophiles the complex *Keap1-Nrf*2 (made up of the transcription factor *Nrf*2 and sensor *Keap*1) is broken and *Nrf*2 is liberated and transported into the nucleus. *Keap*1 has been known to sequester *Nrf*2 in the cytoplasm and also leads to the proteasomal degradation of *Nrf*2. Once the complex is broken, *Nrf*2 is phosphorylated and transported to the nucleus. Inside the nucleus, *Nrf*2 forms heterodimers with small Maf proteins (*SMP*) which then binds to the anti-oxidant response element (*ARE*) and leads to the translation of antioxidant genes, which produces Phase II detoxifying enzymes. The purpose of this is to detoxify the electrophiles to water soluble components. Thus in response to elevated concentrations of electrophiles, various antioxidant proteins are activated [9-13]. The schematic diagram for *Nrf*2 activation is shown in Figure 2. In the rest of this paper, the term ARE will be interchangeably used to represent either the antioxidant response element cis enhancer sequence that is upstream of the gene promoters for the antioxidant proteins or the antioxidant genes/proteins themselves. The context will make it clear whether we are referring to the regulatory sequence or to the resulting gene/protein.

**Figure 3:**
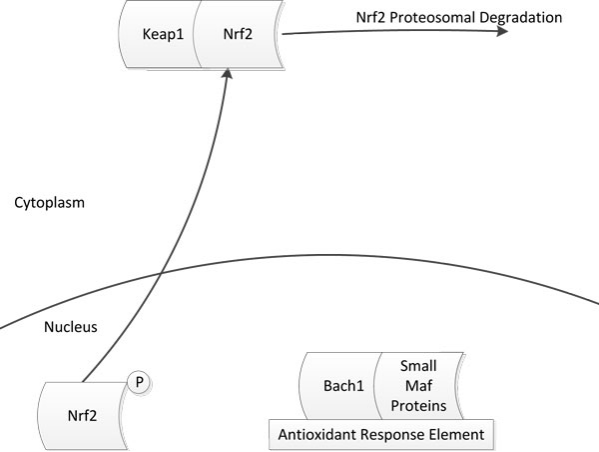
**Nrf2 Deactivation**. Explains how after neutralizing free radicals Nrf2 is transported back to cytoplasm from mitochondria.

We next focus on the procedure by which *Nrf*2 is deactivated. This is carried out by other proteins that stop translation of the antioxidant genes once the electrophiles have been neutralized. For instance, the *Bach*1-*SMP *complex has been known to bind to the same region on the *ARE *as the *Nrf*2-*SMP *complex. Similarly, small Maf proteins are known to form homodimers or heterodimers with other small Maf proteins. These protein complexes are known to bind to the same location on the *ARE *as the *Nrf*2-*SMP *complex. So, once the electrophiles have been eliminated, these protein complexes bind to the *ARE *and displace *Nrf *2 which is then transported back to the cytoplasm. In the cytoplasm, it binds with *Keap*1, which directs its proteosomal degradation [14-17]. The schematic diagram for *Nrf *2 deactivation is shown in Figure 3.

One of the byproducts of normal metabolism is the production of a large number of free radicals. Oxidative stress is caused by the production of free radicals in quantities beyond those that can be handled by the cellular antioxidant system. Indeed, oxidative stress has been implicated in the development of many age-related diseases, including neurodegenerative ones, such as Alzheimer's and Parkinson's, and in aging itself. In addition, excess free radicals react with the nucleotides in the DNA resulting in mutations in the long run. Although there are cellular mechanisms to sense and repair the oxidative DNA damage, mutations can accumulate over a period of time and result in a major disease like cancer. In the next section, we develop a Boolean network model for oxidative stress response pathways. This network will be later utilized to analyze different failure modes that can supress apoptosis and possibly lead to cancer.

### Boolean network modeling of oxidative stress response pathways

Before proceeding to the actual modeling of the specific oxidative stress response pathways, we first formally define the general terms 'pathway' and 'Boolean Network' following the detailed development in [5]. Given two genes/proteins *A *and *B *and binary values *a*, *b *∈ {0, 1}, we define the term *pathway segment *$A\overset{t:a,b}{\to }B$ to mean that if gene/protein *A *assumes the value *a *then gene/protein *B *transitions to *b *in no more than *t *subsequent time steps. A *pathway *is defined to be a sequence of pathway segments of the form $A\overset{t_{1}:a,b}{\to }\;B\overset{t_{2}:b,c}{\to }C$.

A *Boolean Network *(*BN*), ϒ = (*V*, *F *), on *n *genes is defined by a set of nodes/genes *V *= {*x*_1_, ..., *x_n_*}, *x_i _*∈ {0, 1}, *i *= 1, ..., *n*, and a list *F *= (*f*_1_, ..., *f_n_*), of Boolean functions, *f_i_*: {0, 1}*^n ^***→ **{0, 1}, *i *= 1, ..., *n *[18]. The expression of each gene is quantized to two levels, and each node *x_i _*represents the state/expression of the gene *i*, where *x_i _*= 0 means that gene *i *is OFF and *x_i _*= 1 means that gene *i *is ON. The function *f_i _*is called the *predictor function *for gene *i*. Updating the states of all genes in ϒ is done synchronously at every time step according to their predictor functions. At time *t*, the network state is given by *x*(*t*) = (*x*_1_(*t*), *x*_2 _(*t*), ..., *x_n_*(*t*)), which is also called the *gene activity profile *(*GAP*) of the network.

The modeling approach that we will follow here involves using the biological pathway knowledge from the literature and applying Karnaugh map reduction techniques to it to obtain an update equation for each node of the Boolean network [5]. The details specific to the oxidative stress response pathway are discussed next. The pathway segments relevant to the oxidative stress response are given below [9,10,12,15,19,20]:

(1) $$ROS\overset{1:1.0}{\to }K\;eap1$$

(2) $$ROS\overset{1:1.1}{\to }PKC$$

(3) $$ROS\overset{1:a.ā}{\to }Bach\;1$$

(4) $$Keap\;1\overset{1:b.\bar{b}}{\to }Nrf2$$

(5) $$Nrf2,ROS\overset{1:\left(1,0\right),1}{\to }Keap1$$

(6) $$PKC\overset{1:1.1}{\to }Nrf2$$

(7) $$Bach1,SMP\overset{1:\left(1,1\right),0}{\to }ARE$$

(8) $$Nrf2,SMP\overset{1:\left(1,1\right),1}{\to }ARE$$

(9) $$SMP,SMP\overset{1:\left(1,1\right),0}{\to }ARE$$

(10) $$ARE\overset{1:1,1}{\to }SMP$$

(11) $$ARE\overset{1:1,0}{\to }ROS$$

(12) $$ARE\overset{1:1,0}{\to }PKC$$

**Figure 4:**
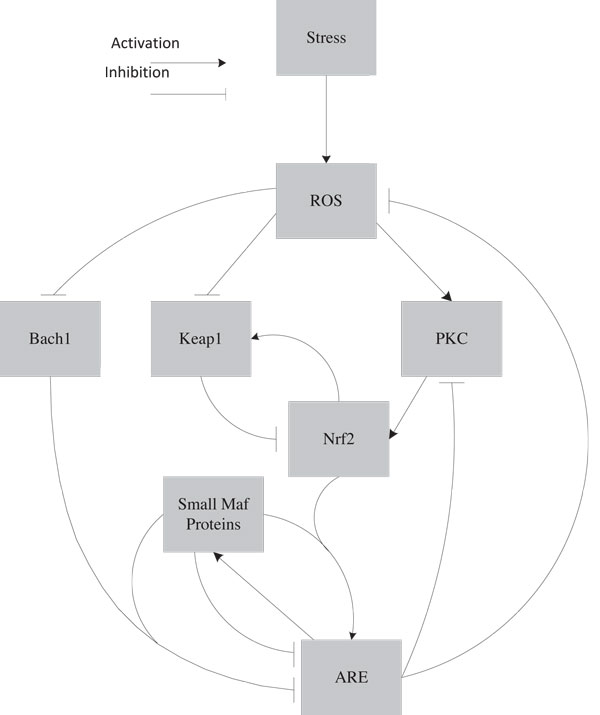
**Oxidative Stress Response Pathways**. The major pathways involved in oxidative stress response.

**Figure 5:**
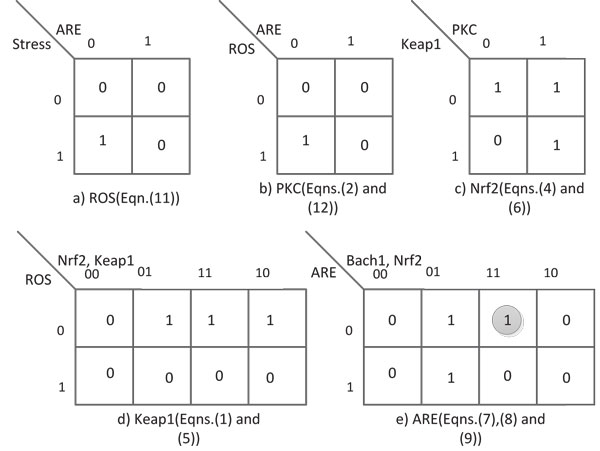
**Karnaugh Maps for Deriving the Oxidative Stress Response Boolean Network**. K-map simplification for all the elements involved in the system.

In these pathways *ARE *represents the family of antioxidant genes in the sense that if the correct complexes bind to *ARE *it leads to the up-regulation/down-regulation of the appropriate antioxidant gene. These pathway interactions are pictorially represented in Figure 4. In this figure we have used square boxes without making any distinction between whether they represent proteins/genes or a biochemical entity. *ROS *stands for reactive oxidative species which is a biochemical entity. The other entities like *PKC*, *Keap*1, *Nrf*2, *Bach*1 are all proteins and *ARE *(Antioxidant Response Element) is a cis enhancer sequence that is upstream of the gene promoters for the antioxidant proteins or the antioxidant genes/proteins themselves. Also the merged activation (*Nrf*2/*SMP*) or inhibition (*Bach*1/*SMP*) corresponds to dimers formed between these components. The Karnaugh-maps for the genes/proteins are shown in Figure 5.

From the pathways described above and using the Karnaugh-map reduction techniques, the Boolean update equations for each node of the network are deduced. Some logical reasoning has been used for determining the equations: 1) the maximum number of predictors for updating a variable is fixed to be 3; 2) Small Maf Protein is assumed to be ubiquitously expressed and the pathway given by Eqn.(10) only increases the concentration of *SMP*, which in conjunction with Eqn.(9), binds to *ARE *and down-regulates the antioxidant gene; 3) a gene being turned on implies that the corresponding protein is being produced although, in reality, this is not necessarily true; and 4) in the case of a conflict in the Karnaugh map, biological knowledge has been used to assign either a 0 or a 1. This last point is demonstrated by a specific example. For instance, in the case of *ARE*, the entry shown with a grey circle around it says that when both *Bach*1 and *Nrf*2 are upregulated and antioxidant gene is downregulated, then at the next time step antioxidant gene will be upregulated. The biological explanation for such an update is that it corresponds to the situation where, in the presence of *Stress*, *Nrf*2 has been activated and is relocating to the nucleus while the inhibitor *Bach*1 is simultaneously relocating to the cytoplasm prior to the activation of antioxidant gene at the next time step. Such intuitive reasoning has been used to model the system here. One might use a different reasoning which could lead to a different set of update equations. However, since we are concerned only about the final steady-state behavior, such reasoning can be justified as long as the overall system behavior, defined by the update equations, matches the steady-state. As an example, the final update equation for *ARE *is derived as follows. In the K-maps, the ones are grouped up in pairs of 2,4,8 and so on and each group should have at least one variable staying constant. So for this case there are two groups whose equations correspond to $Nrf2\cdot \left(\bar{ARE}\right)$ and $Nrf2\cdot \left(\bar{Bech1}\right)$. The final update equation for *ARE *is the sum of these two equations. Please refer to Additional file 1 for some additional details. Indeed, by working with different sets of update equations, we determined that all biologically plausible ones led to the same/similar attractor behavior. From the set of possible Boolean networks we chose the ones that appealed most to our biological understanding and the resulting update equations are given below:

(13) $$ROS_{next}=Stress\cdot \bar{ARE}$$

(14) $$Keap1_{next}=\bar{ROS}\cdot \left(Nrf2+Keap1\right)$$

(15) $$PKC_{next}=ROS\cdot \bar{ARE}$$

(16) $$Nrf2_{next}=PKC+\bar{Keap1}$$

(17) $$Bach1_{next}=\bar{ROS}$$

(18) $$SMP_{next}=1$$

(19) $$ARE_{next}=Nrf2\cdot \left(\bar{ARE}+\bar{Bach1}\right).$$

**Figure 6:**
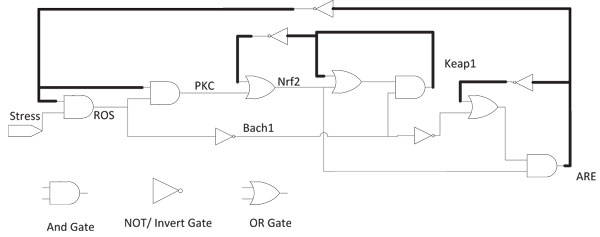
**Equivalent Boolean Network for Oxidative Stress Response**. Boolean network model for oxidative stress response based on the equations derived using K-maps.

**Figure 7:**
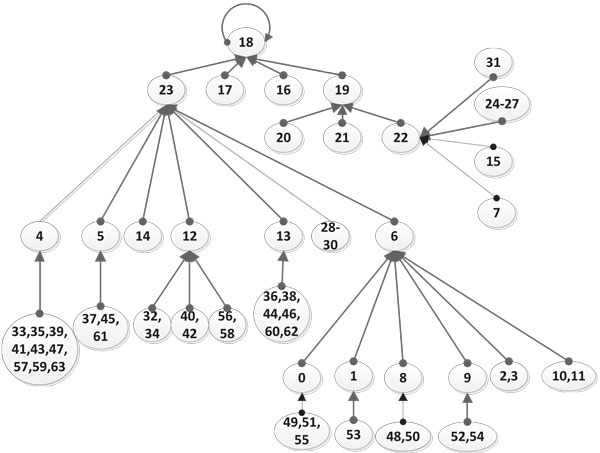
**The Boolean State Transition Diagram when the Stress input is 0**. The state transition diagram for the Boolean network with no stress on the system. This gives us an idea of the attractor states of the system.

**Figure 8:**
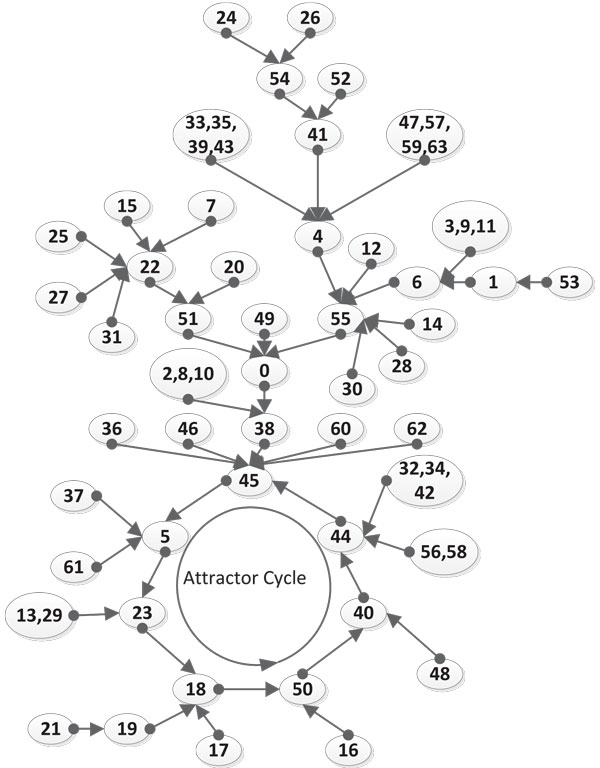
**The Boolean State Transition Diagram when the Stress input is 1**. The state transition diagram for the Boolean network with stress on the system. This gives us an idea of the attractor states of the system.

An equivalent digital circuit with logic gates is shown in Figure 6. Here the lines in bold represent feedback paths. The state transition diagrams resulting from Eqns. (13)-(19) for the two cases *Stress *= 0 and *Stress *= 1 are shown in Figures 7 and 8 respectively. In these transition diagrams, the genes in the binary state representation are ordered as [*ROS Keap*1 *PKC Nrf*2 *Bach*1 *ARE*] and the binary states are compactly represented by their decimal equivalents. For instance, the binary state (111100) would be represented by the decimal number 60. The states of particular interest are the attractors as they give rise to the steady-state properties of the network. In Figure 7, the state of interest is the singleton attractor 18(010010). On the other hand, in Figure 8, the states of interest are the seven states forming the attractor cycle. These states are: 18(010010), 50(110010), 40(101000), 44(101100), 45(001101), 5(000101) and 23(010111) traversed in that order. They would lead to cyclical/oscillatory behavior in the time domain response.

It is clear from the preceding discussion that some kind of oscillatory behavior of the genes will be observed when the external *Stress *input equals 1. On the other hand, when the *Stress *input equals 0, the system will rest in only one state meaning that there will be no oscillation.

### Time domain simulation results

**Figure 9:**
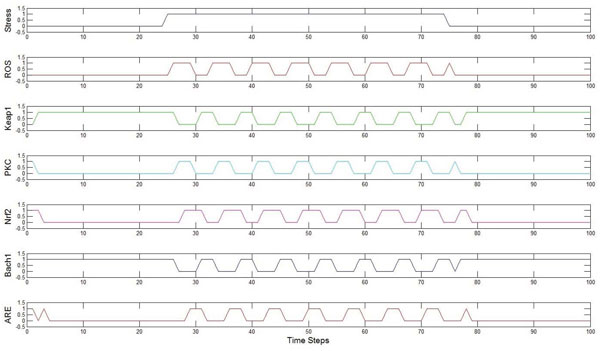
**Time response behaviour of the system in Fig.4**. Time response simulation of the Boolean network to observe oscillations of the proteins in the system.

The network obtained was simulated using MATLAB by giving an external stress input signal for a duration of 50 *timesteps*, and both the input signal and the responses are shown in Figure 9. The signal *ROS *is a biological manifestation of the external input signal, *Stress *being applied to the network. The biological purpose of this network is to counteract the effect of *ROS *produced in response to the *Stress *input. As we can see from Figure 9, in the absence of any *Stress *signal, the system reaches the singleton attractor 18(010010). Once *Stress *signals are applied, there are oscillations as theoretically expected from the existence of an attractor cycle. In Reichard *et al. *[14], the cells were treated with Arsenite, a well known activator of *Nrf*2 and an out-of-phase relationship was observed between *Nrf*2 and *Bach*1. Shan *et al. *[17] also showed a similar out of phase relationship. In Katsuoka *et al. *[16]*DEM *(an activator of *Nrf*2) also leads to increased expression of *NQO*1 which is a known anti-oxidant response element. Such an in-phase relationship between *Nrf*2 and the antioxidant gene is also seen in Figure 9. Thus the theoretical predictions made by our Boolean network model for oxidative stress response appear to be consistent with experimental observations from the published literature. Note, however, that these experiments consider only two genes/proteins at a time and therefore, there is a need for experimentally studying the simultaneous activities of *ROS*, *Keap*1, *Nrf*2, *PKC*, *Bach*1 and *ARE *in the time domain.

### Mitochondria and free radical generation

**Figure 10:**
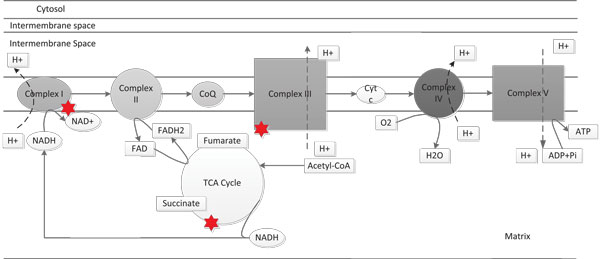
**Stages of Oxidative Phosphorylation producing free radicals**. Explains Krebs cycle and how and where free radicals are produced in the mitochondria.

Mitochondria play an important role in cellular energy metabolism, free radical generation and apoptosis. It has long been suspected that mitochondrial functions contribute to the development and progression of cancer [21-23]. Over 70 years ago, Otto Warburg proposed that a key event in carcinogenesis is a defect in the respiratory mechanism, leading to increased glycolysis even in the presence of oxygen;this is known as the Warburg effect [24]. The well known function of mitochondria is to generate Adenosine Triphosphate (*ATP*) molecules providing energy for the survival of the cell through oxidative phosphorylation (*OXPHOS*), which is collectively accomplished by proteins encoded both by nuclear and mitochondrial DNA. Oxidative phosphorylation is a metabolic process, which takes place in mitochondria in which *ATP *is formed as a result of the transfer of electrons from *NADH *or *FADH*_2 _to *O*_2 _by a series of electron carriers. *OXPHOS *is the major source of *ATP *as well as free radical generation in aerobic organisms. For example, oxidative phosphorylation generates 26 of the 30 molecules of *ATP *that are formed when a molecule of glucose is completely oxidized to *CO*_2 _and *H*_2_*O*, although 1 to 2% of the electrons are lost during transfer through the chains leading to free radical generation [25]. Figure 10[26] shows a schematic representation of the whole process along with free radical generation. The points shown with red stars correspond to the locations where free radicals are generated.

Even though it has been long recognized that increased *ROS *production in mitochondria leads to genetic instability and progression of cancer, there remain several unanswered questions regarding the complex signalling capacity of this organelle [27]. The DNA is highly susceptible to free radical attacks. Free radicals can break DNA strands or delete bases. These mutations can prove to be carcinogenic. It has been estimated that more than 10,000 hits of oxidative stress occur each day. So it is important to tackle these free radicals at the source of their generation, which is why the mitochondria is also a very rich source of anti-oxidants. Although cellular mechanisms can tackle this stress, damage accumulates with age. At present altered energy metabolism is considered to be an additional hall mark of cancer progression [28] and these metabolic pathways have been investigated as targets for cancer therapy. In this paper, we will specifically focus on the *PI*3*k*/*Akt *pathway which is one such pathway and is described in the following section.

### An integrated network for oxidative stress response and apoptosis

**Figure 11:**
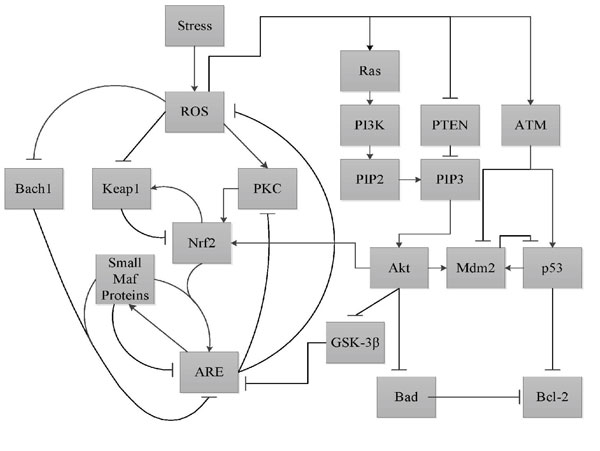
**Pathway Diagram of Oxidative Stress along with PI3k/Akt**. Inclusion of PI3k/Akt pathways along with oxidative stress pathways and study how they can lead to aberrant be-haviour in cells.

**Figure 12:**
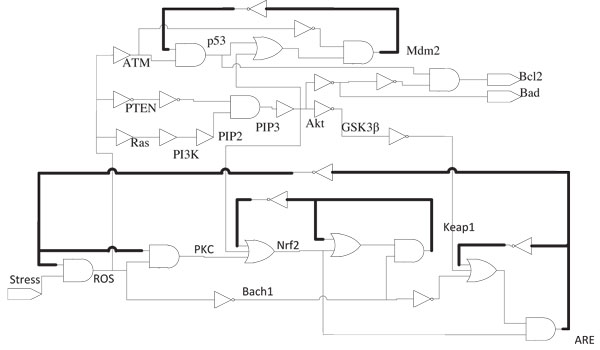
**Boolean Network modeling of Fig. 11**. A Boolean network model of the network along with PI3k/Akt pathways.

Cancer is an umbrella term for diseases that are associated with loss of cell-cycle control, leading to uncontrolled cell proliferation and/or reduced apoptosis. It is often caused by genetic alterations leading to malfunctioning in the biological pathways [1,29,30]. One of the possible cellular responses resulting from oxidative stress is the induction of apoptosis. Thus it is important to develop a network model linking the oxidative stress input to the fate of the cell. In this section, we will do precisely that by considering the oxidative stress response pathways alongside other downstream pathways capable of inducing apoptosis. Specifically, we will focus on the *PI*3*k*/*Akt *pathway. The *PI*3*k*/*Akt *pathway is downstream of the *Ras *gene which is known to play an important role in many cancers. In addition, other genes in the *PI*3*k*/*Akt *pathways are found mutated in many cases of cancer. Oxidative stress often upregulates many of the genes in the *PI*3*k*/*Akt *pathway. The detailed interactions between the oxidative stress response pathway and the *PI*3*k*/*Akt *pathway are shown in Figure 11[1,31-33]. Starting with this pathway diagram and utilizing the procedure developed earlier in Section, an equivalent digital circuit in terms of logic gates can be implemented as shown in Figure 12. The above circuit is modeled with two output genes which effectively control the final fate of the cell. *Bad *and *Bcl*2 are known to have pro-apoptotic and anti-apoptotic functions respectively and thus can serve as biomarkers of apoptosis induction. Indeed, it is the delicate balance between the activity of these two genes that dictates the ultimate fate of the cell [34-36]. The purpose of the *Nrf*2-*ARE *pathway in this integrated network is to reduce the average value of *ROS *present in the system, in response to the oxidative stress. This is clear from the plot in Figure 9: between the time instants from the 25*^th ^timestep *to the 75*^th ^timestep *when there is a continuous *Stress *present in the system, the *ROS *present in the system is oscillating between 0 and 1 which implies that its average value is less than '1', which is the value that we would have otherwise had in the absence of the *Nrf *2-*ARE *pathway.

### Classification of faults in the integrated network

In the integrated pathway diagram of Figure 11, the two genes namely *Bad *and *Bcl*2 are instrumental in deciding the fate of the cell. The preferred status of the two genes, when oxidative stress is not being neutralized, are 1 and 0 respectively since it corresponds to the situation where the pro-apoptotic factor is turned ON and the anti-apoptotic factor is turned OFF. Although a deviation from this state may not signal that the cell is turning cancerous, there is a higher possiblity of the cell exhibiting aberrant behaviour.

Depending on the final resting status of these two genes, one may be able to characterize the degree of invasiveness of the disease especially if it is being caused by apoptosis supression. Once it has been determined that a cell is exhibiting abberant behavior, one would like to pinpoint the location of the fault/error so that the necessary therapeutic intervention(s) can be applied. Since the digital circuit model of Figure 12 uses logic gates, it should be possible to use the fault detection techniques from the Digital Logic literature [37,38] to pinpoint the fault locations. This will be carried out in this section. An important difference between the results obtained in Layek *et al. *[6] for pinpointing the fault locations in the *MAPKinase *pathways and the results to be presented here is that the digital circuit in Figure 12 involves feedback and its behaviour is, therefore, much more complicated to analyze. However, it should be pointed out that the simpler fault pinpointing methodology presented in Layek *et al. *[6] is much more amenable to biological validation via appropriately designed experiments while the same cannot be said about the results to be presented here. Indeed, the results to be presented here show that the pinpointing of the fault locations is theoretically possible even in this case, although the biological feasibility of the methods required is open to question.

We note that the faults in a digital circuit are mainly of two types [37]:

• Stuck-at Faults: As the name implies, this is a fault where a particular line *l *is stuck at a particular value α ∈ {0, 1}, denoted by line *l*,s-*a*-*α *(*s-a*-*α *means stuck-at-α). This means that the value at that line is always going to be *α *regardless of the inputs coming in. This can be thought of as something similar to a mutation in a gene, where a particular gene is either permanently turned ON or OFF.

• Bridging Faults: This is the type of fault where new interconnections are introduced among elements of the network. This can be thought of as new pathways being created in the cell. This type of fault is not considered in the current paper due to the lack of biological knowledge about new pathways being introduced.

Here, it is appropriate to mention that the biological relevance of each of these two types of faults has been discussed in Layek *et al. *[6].

The digital circuit in Figure 12 has feedback (shown in bold lines) and is, therefore, a sequential circuit. To detect a fault in a sequential circuit we need a test sequence. Let *T *be a test sequence and let *R*(*q,T*) be the response of the fault-free sequential system *N *starting in the intial state *q*. Now let the faulty sequential circuit be denoted by *N_f _*where *f *is the fault. Let us denote by *R_f_*(*q_f_,T*) the response of *N_f _*to *T *starting in the initial state *q_f_*. A test sequence *T *detects a fault *f *iff (if and only if or equivalently this condition is both necessary and sufficient) for every possible pair of initial states *q *and *q_f_*, the output sequences *R*(*q,T*) and *R_f _*(*q_f _*,*T*) are different for some specified vector *t_i _*∈ *T*. The output being observed is the status of [*Bad*, *Bcl*2].

**Figure 13:**
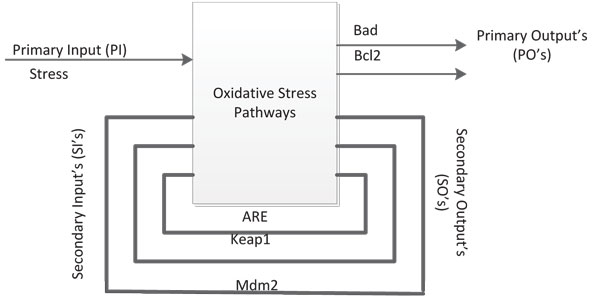
**Block Diagram Representation of Fig.12**. A simple description of the system showing clearly the feedback lines in the system.

Once this output shows a deviation from a desired value, it becomes imperative to pinpoint the possible fault locations which can give rise to the aberrant behaviour. To do so, one can represent the digital circuit of Figure 12 as in Figure 13. The Primary Input(PI) is *Stress *which is the only external signal which the experimenter has control over. The Primary Output's (PO's) are the status of *Bad *and *Bcl*2, which are the only outputs available to the experimenter. The Secondary Output's and Secondary Input's are [*ARE, Keap*1*, Mdm*2], which are being fed back into the system. The states of these 3 genes *ARE, Keap*1 and *Mdm*2 determine the internal state of the system. These 3 elements can be considered as memory elements of the system as their previous state is retained by the system and fed back. The input sequence consists of two parts namely a Homing sequence and a Test sequence, denoted by *H *and *T *respectively.

The purpose of this procedure is to pinpoint the possible locations for the fault *f *in *N_f_*, given the output sequence of *Bad *and *Bcl*2 for the normal and faulty circuits. It is assumed that we have no knowledge about the initial status of any of the genes. Knowledge of the initial status of the internal states is important as all future computations are based on these values. The Homing sequence is an initial input sequence that brings the network to a known internal state. So, once the Homing Sequence is given to *N *and *N_f_*, *N *will come to a known internal state. Note that a similar claim cannot be made about *N_f _*as the fault *f *is not known apriori. For the circuit in Figure 13, a possible Homing sequence is [0 0 0 0 0 0 0 0], which brings the internal state of the system to [0 1 0]. This means that if the *Stress *input is zero for eight time steps, then at the end of that period, the internal state of the system becomes [0 1 0], regardless of the initial status of any of the genes in the network. A reason for choosing this Homing sequence is that it implies that no input needs to be given to the system and it evolves to the indicated internal state. In future when we are trying to validate these results experimentally this will be of immense help. If we refer back to Figure 7, we see that regardless of the initial state, within four time steps the trajectory reaches the state ('010010') where *ARE *= 0 and *Keap*1 = 1. This is consistent with the conclusion that we are getting from the Homing sequence here with the only difference that a slightly longer sequence is required here as the state transition diagram has a higher cardinality than that in Figure 7.

**Figure 14:**
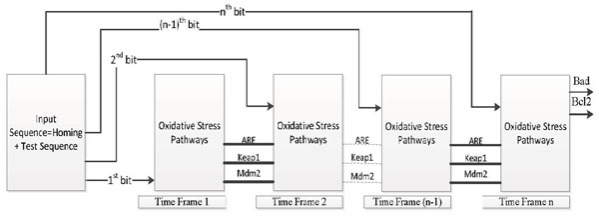
**Fault Detection using Time-Frame Expansion**. Fault detection in the boolean(digital) network using time-frame expansion method.

Once the Homing sequence has done its job, the Test sequence(*T*) is fed into *N *and *N_f_*, and by comparing the output states of the normal and faulty networks, we can pinpoint the location of the fault in the network, assuming that a single stuck-at-fault has occurred. This can be carried out using the *time-frame expansion method *which is briefly discussed next. The block in Figure 13 is replicated *n *times with the feedback loops cut-off. The Secondary Output of the *k^th ^*stage is fed as the Secondary Input for the (*k *+ 1)*^th ^*stage. The Primary Outputs of the first (*n *- 1) stages are neglected. The Primary Outputs of the *n^th ^*stage of the normal and faulty circuits will be different as the network configurations are different for both. The Primary Input sequence has to be derived so that the error in a line is propagated to the primary output in *n *time steps, so that a difference is observed at the primary outputs of the normal and faulty circuits [37,38]. The situation is pictorially represented in Figure 14. Please refer to Additional file 1 for to y example.

**Figure 15:**
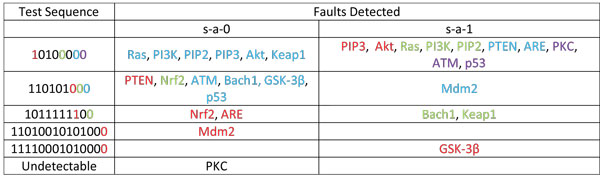
**Test Sequences for detecting single *stuck-at-faults***. The test sequence which can be given to system to find out single stuck-at-faults based on output signature.

From the preceding discussion in this section, we know that there are 15 possible genes (this is the total number of genes in Figure 11, excluding the output genes *Bad *and *Bcl*2) where there could be a mutation. This means that there are 30 cases of faults as a single gene can be mutated as a *s-a*-0 or as a *s-a*-1. We consider all possible cases of single mutation, because in the presence of mutation, the normal and faulty system cannot produce the same output unless, of course, the mutated gene is not a critical one. Based on the methods described earlier in this section, we came up with a list of test sequences for the detection of each gene fault. It is to be noted that the Test Sequences generated here are only for the Homing Sequence considered earlier. For a different Homing Sequence the Test Sequence will also be different. The different test sequences and their ability to detect different single stuck-at faults are tabulated in Figure 15. Here, truncated versions of the same test sequence can be used to detect different faults appearing in the same row. For detecting any particular fault, one would apply the test sequence from the same row truncated at the bit whose color matches that of the particular fault. The mismatch between the outputs of the normal and faulty systems, characterized by the vector [*Bad*, *Bcl*2] would then result in the detection of that fault. Thus we have developed a method to pinpoint the possible fault locations in a Boolean network with feedback. The algorithm will work with multiple fault cases too with minor modifications.

## Concluding remarks

In this paper, we have developed a Boolean network model for the oxidative stress response. This model was developed based on pathway information from the current literature pertaining to oxidative stress. Where applicable, the behaviour predicted by the model is in agreement with experimental observations from the published literature. It is our hope that some of the additional predictions here, such as those pertaining to the oscillatory behaviour of certain genes in the presence of oxidative stress, will be experimentally validated in the near future.

We have also linked the oxidative stress response to the phenomenon of apoptosis via the *PI*3*k*/*Akt *pathway. An integrated model based on collectively considering the *PI*3*k*/*Akt *pathways and the oxidative stress response pathways was developed and then used to pinpoint possible fault locations based on the *Bad*-*Bcl*2 apoptotic signatures in response to 'test' oxidative stress inputs. The approach used to achieve this differs significantly from the earlier results in Layek *et al. *[6] since the Boolean network of this paper has feedback. The approaches used here and in Layek *et al. *[6] could potentially have a significant effect on cancer therapy in the future as pinpointing the possible fault location(s) in cancer could permit the choice of the appropriate combination of drugs (such as kinase inhibitors) for maximum therapeutic effectiveness. Of course, it should be pointed out that the theoretical procedure presented here for pinpointing fault locations in a biological network with feedback will need to be further simplified before it can be even considered for practical biological validation.

## Competing interests

The authors declare that they have no competing interests.

## Authors' contributions

Sriram Sridharan did most of the theoretical work on this paper with some assistance from Ritwik Layek. Aniruddha Datta provided overall direction and supervision while Jijayanagaram Venkatraj provided the relevant supporting biological domain knowledge.

## Supplementary Material

Additional file 1 **Explains the algorithms discussed in the manuscript with toy examples**. Click here for file
