# Supplementary material for: Boolean modeling and fault diagnosis in oxidative stress response
Source: BMC Genomics. 2012 Oct 26;13(Suppl 6):S4. doi: 10.1186/1471-2164-13-S6-S4 (PMC3481480; doi:10.1186/1471-2164-13-S6-S4)
Supplement: Additional file 1 — Explains the algorithms discussed in the manuscript with toy examples. [file 1471-2164-13-S6-S4-S1.pdf]

Additional file 1

Arriving at a Test Sequence

As an example on how to obtain the test sequence let us consider the sequential circuit shown below:

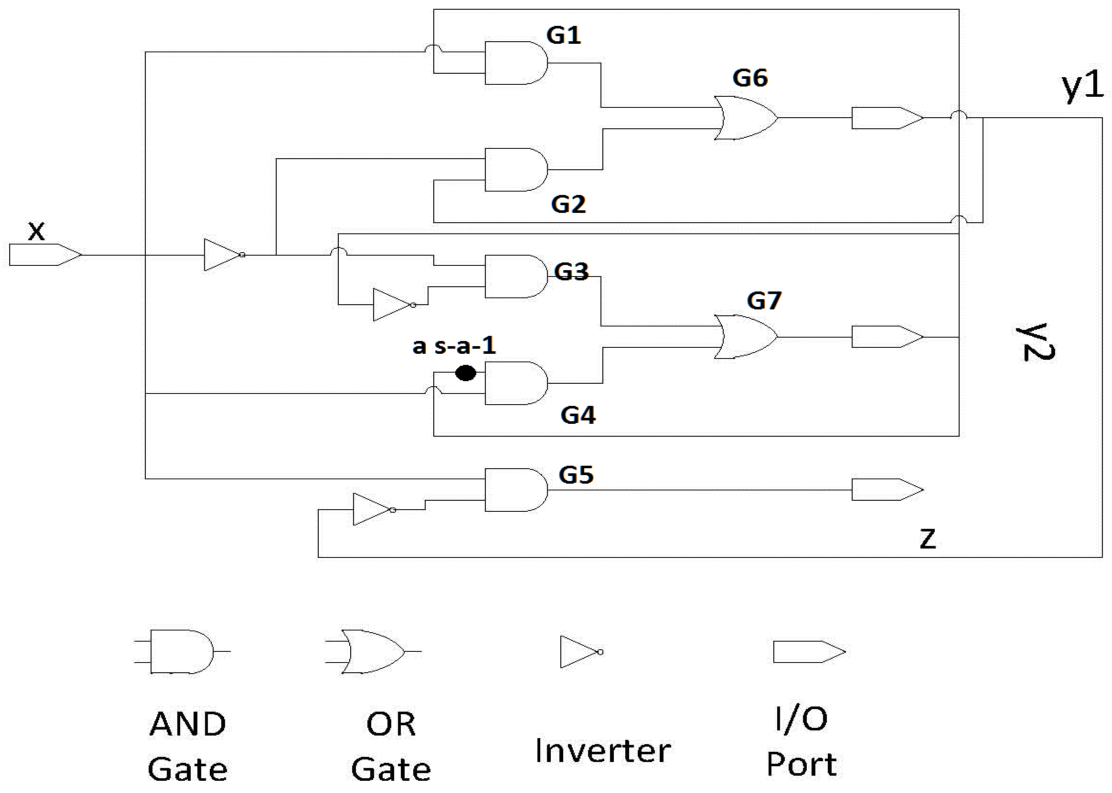

Let us also assume that the internal state is known and is y1=y2=0. Let us determine the test sequence that will determine the fault at location 'a' s-a-1.

Time frame 0: Since y2=0 and we have the line s-a-1. As we need to propagate this fault we choose x=1 so that the output of G4 is going to be 0 for normal circuit and 1 for faulty circuit. The internal state is y1=0, y2=0/1. In the representation a/b, a corresponds to value in normal and b corresponds to value in fault network. The output is z=1.

Time frame 1: Since the state y1 did not change we cannot produce a difference in the output between normal and faulty network so we maintain x=1 and push the system to internal state y1=0/1, y2=0/1 and x=1.

Time frame 2: Since there is difference in state y1 between normal and faulty circuit we can propagate this to z by choosing x=1 and z=1/0.

Hence the test sequence x='111' will determine the fault at a s-a-1.

Solving K-map (equations 13-19)

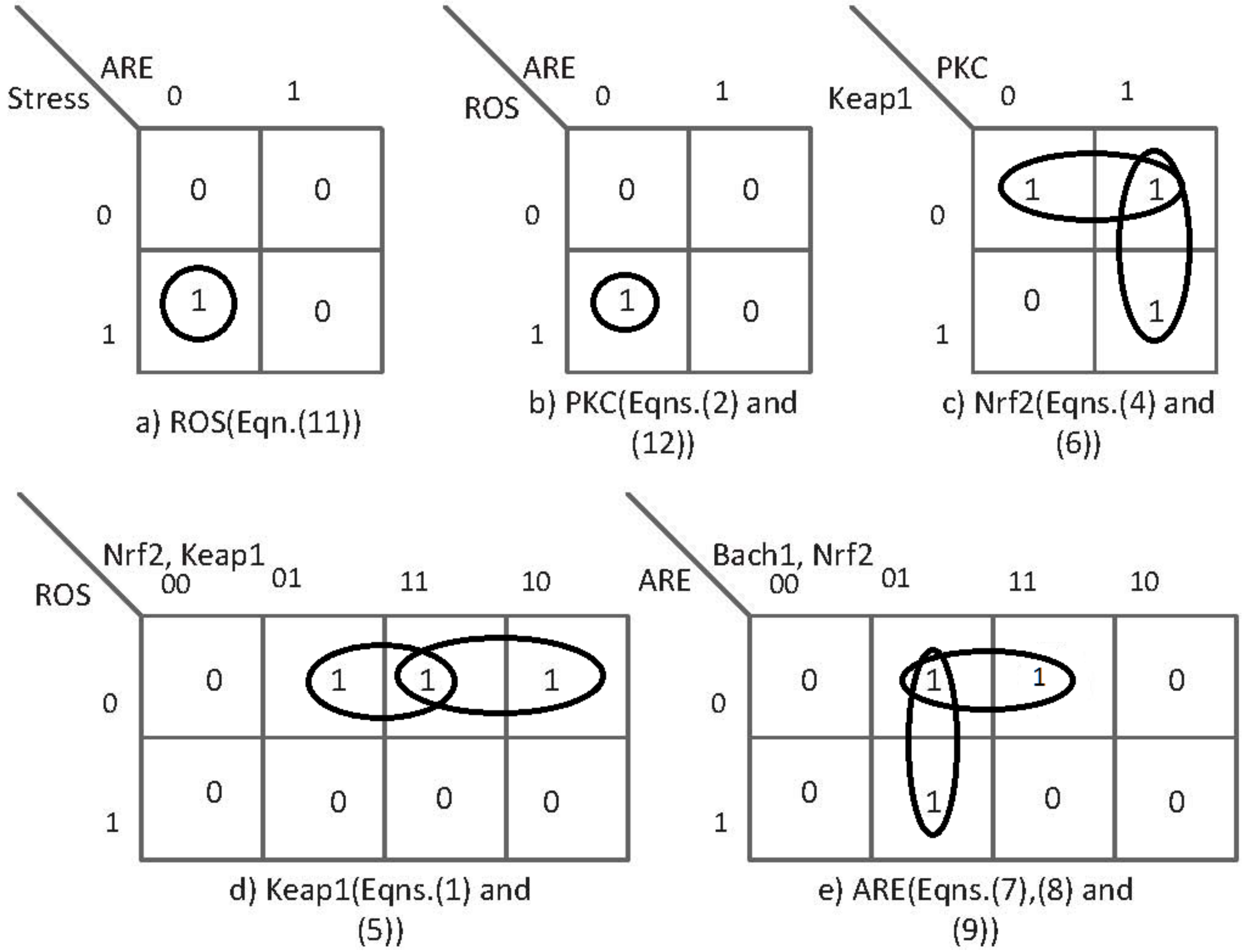

The Karnaugh map (K-map for short), is a method to simplify Boolean algebra expressions. Once K-map has been constructed, the ones (or zeros) can be grouped together. Here we group the ones together.

If we look at K-map for ROS it has only one '1'. So for this to be satisfied $ROS = Stress * \overline{ARE}$. Similarly grouping the ones in other K-maps will yield the other equations. The way the ones needs to be grouped are also shown in the diagram.

Details of the components Fig.4 and Fig.11

Bach1: BTB and CNC homology 1, basic leucine zipper transcription factor 1

Nrf2: Nuclear factor (erythroid-derived 2)-like 2

PKC: Protein Kinase C

ROS: Reactive Oxygen Species

Keap1: Kelch-like ECH-associated protein 1

ARE: Anti-oxidant Response Element

Ras: Rat Sarcoma

PI3K: Phosphoinositide 3-kinase

PIP2: Phosphatidylinositol 4,5-bisphosphate

PIP3: Phosphatidylinositol (3,4,5)-trisphosphate

PTEN: Phosphatase and tensin homolog

ATM: Ataxia Telangiectasia Mutated

Akt: Protein Kinase B

Mdm2: mouse double minute 2

p53: Tumor protein 53

GSK-3β: Glycogen synthase kinase 3 beta

Bad: BCL2-associated agonist of cell death

Bcl-2: B-cell lymphoma 2
